# Evidence for sodium valproate toxicity in mitochondrial diseases: a systematic analysis

**DOI:** 10.1136/bmjno-2024-000650

**Published:** 2024-06-05

**Authors:** Thiloka E Ratnaike, Nour Elkhateeb, Angela Lochmüller, Christopher Gilmartin, Katherine Schon, Rita Horváth, Patrick F Chinnery

**Affiliations:** 1 Department of Paediatrics, University of Cambridge, Cambridge, UK; 2 Department of Paediatrics, Colchester Hospital University NHS Foundation Trust, Colchester, UK; 3 Department of Clinical Genetics, Cambridge University Hospitals NHS Foundation Trust, Cambridge, UK; 4 Department of Medical Genetics, Cambridge Biomedical Campus, Cambridge, UK; 5 University College London Hospitals NHS Foundation Trust, London, UK; 6 University of Nottingham, Nottingham, UK; 7 Cambridge Biomedical Campus Department of Clinical Neurosciences, University of Cambridge, Cambridge, Cambridgeshire, UK; 8 Medical Research Council Mitochondrial Biology Unit, Cambridge Biomedical Campus, Cambridge, UK

**Keywords:** MITOCHONDRIAL DISORDERS, NEUROGENETICS, NEUROPHARMACOLOGY, EPILEPSY, GENETICS

## Abstract

**Background:**

We aimed to determine whether sodium valproate (VPA) should be contraindicated in all mitochondrial diseases, due to known VPA-induced severe hepatotoxicity in some mitochondrial diseases.

**Methods:**

We systematically reviewed the published literature for mitochondrial DNA (mtDNA) and common nuclear genotypes of mitochondrial diseases using PubMed, Ovid Embase, Ovid Medline and MitoPhen databases. We extracted patient-level data from peer-reviewed articles, published until July 2022, using the Human Phenotype Ontology to manually code clinical presentations for 156 patients with genetic diagnoses from 90 publications.

**Results:**

There were no fatal adverse drug reactions (ADRs) in the mtDNA disease group (35 patients), and only 1 out of 54 patients with a non-*POLG* mitochondrial disease developed acute liver failure. There were fatal outcomes in 53/102 (52%) *POLG* VPA-exposed patients who all harboured recessive mutations.

**Conclusions:**

Our findings confirm the high risk of severe ADRs in any patient with recessive *POLG* variants irrespective of the phenotype, and therefore recommend that VPA is contraindicated in this group. However, there was limited evidence of toxicity to support a similar recommendation in other genotypes of mitochondrial diseases.

**What is already known on this topic**

- Sodium valproate (VPA) is known to cause severe hepatotoxicity in patients with mitochondrial diseases caused by *POLG* variants, and therefore, is often avoided in patients with other genetic causes of mitochondrial diseases.

**What this study adds**

- This review confirmed that VPA should be contraindicated in all patients with mitochondrial diseases caused by recessive *POLG* variants, however, there was limited evidence to support the same recommendation for other genotypes of mitochondrial diseases. Patients with epilepsy caused by mitochondrial DNA diseases, treated by VPA, were not found to have fatal adverse drug reactions.

**How this study might affect research, practice or policy**

- This review suggests that further natural history studies are required in patients with non-*POLG* mitochondrial diseases, and that clinicians should consider requesting rapid genomic testing in children with early-onset epilepsies where *POLG*-mitochondrial disease is a differential diagnosis, prior to commencing VPA.

## Background

Epilepsy often features in mitochondrial diseases, with myoclonic, generalised and focal onset seizure types.^1^ Sodium valproate (VPA) is effective in these seizure types in non-mitochondrial diseases, although not a first-line treatment for focal onset seizures.^2^ It is also used for migraines and bipolar disorder prophylaxis, which are found in mitochondrial diseases.^3–5^ However, it is important to note that VPA has known teratogenic effects and associations with neurodevelopmental disorders, leading it to only be considered in the event of a lack of alternative options.^6^ While VPA may cause transient liver enzyme increases in 15% of patients, severe adverse drug reactions (ADRs) like hepatic failure are rarer (0.01% of all patients).^7^ *POLG*-related mitochondrial disease, typically Alpers syndrome presenting with developmental regression and intractable seizures, is an identified risk factor for VPA-induced hepatotoxicity.^7^ *POLG* disease can present at different ages with seizures commonly presenting in early-onset and juvenile to adult-onset forms. However, liver involvement is prevalent in early-onset *POLG* disease and is associated with worse survival including in phenotypes where epilepsy is not a feature.^8^ The severity of VPA-related ADRs in Alpers syndrome and the lack of systematic follow-up studies of VPA in other mitochondrial diseases have led clinicians to avoid VPA in all mitochondrial diseases without clear evidence, potentially discarding an effective and cheap medicine. To bridge this gap, we assessed VPA effects across various mitochondrial diseases. MitoPhen,^9^ which contains published clinical data as human phenotype ontology (HPO) terms, was used to investigate whether there are any associated clinical features pre-empting VPA-related toxicity. Our aim was to evaluate the evidence base for VPA-induced toxicity in different mitochondrial diseases using published patient-level data.

## Methods

### Search strategy and study selection

**Figure 1:**
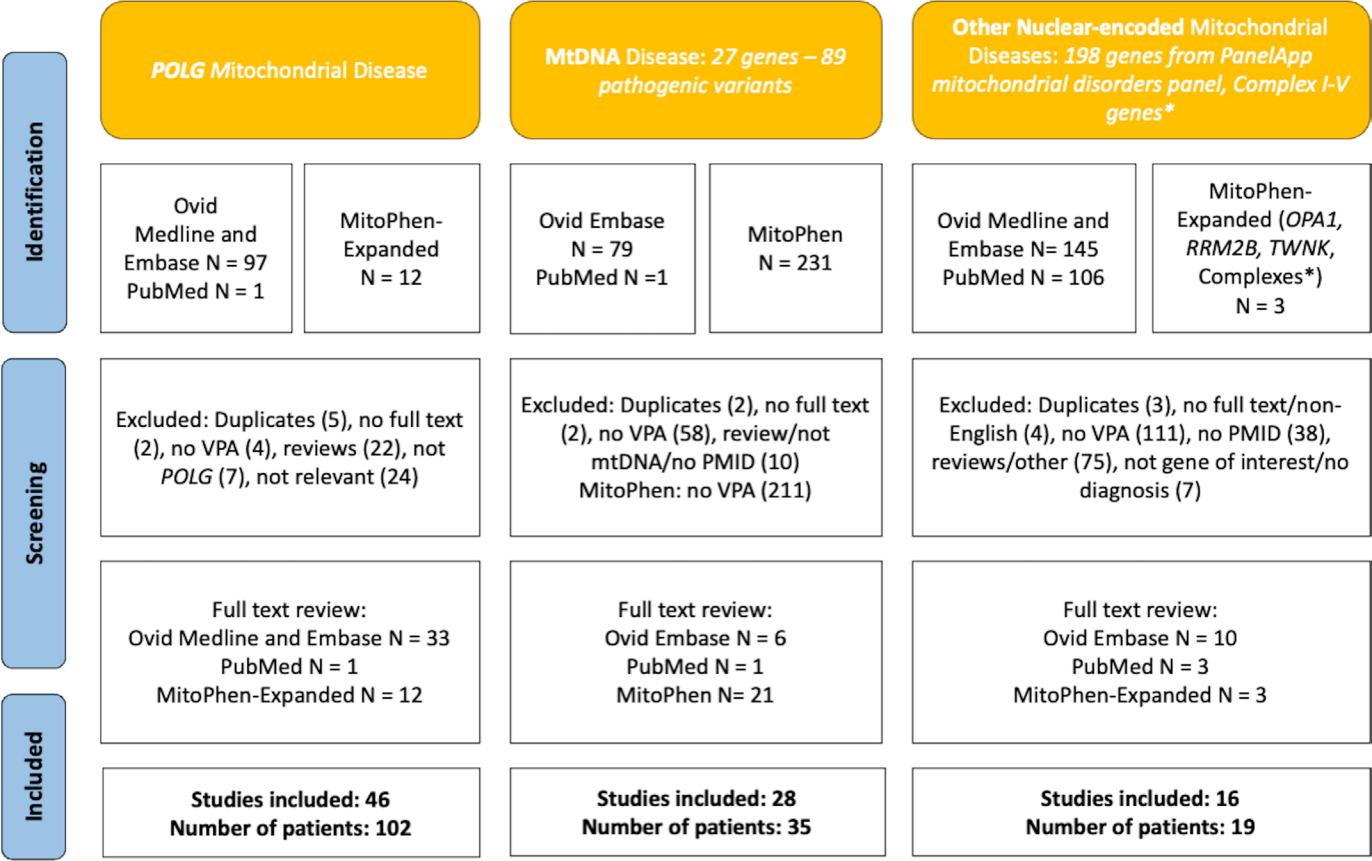
Preferred Reporting Items for Systematic Reviews and Meta-analyses approach to study inclusion in systematic review. Searches were performed separately for *POLG*-related mitochondrial disease, mtDNA disease, other nuclear-encoded mitochondrial diseases. *Nuclear genes associated with complex I-V deficiencies were only searched in the MitoPhen-Expanded data set. mtDNA, mitochondrial DNA; PMID, PubMed Identifier; VPA, valproate.

A systematic review of the literature, published up to July 2022, was conducted using PubMed, Ovid Embase, Ovid Medline and MitoPhen databases, according to the Preferred Reporting Items for Systematic Reviews and Meta-analyses^10^ (figure 1). Articles about mitochondrial diseases linked to migraine or seizures and VPA treatment were reviewed. We considered three groups: (1) *POLG-*related mitochondrial diseases, (2) mitochondrial DNA (mtDNA) diseases and (3) other nuclear causes of mitochondrial disease based on PanelApp genes.^11^ We supplemented the search with the ‘MitoPhen-Expanded’ data set which currently contains data on 89 pathogenic mtDNA variants,^9^ and common nuclear genotypes including *POLG*. Exclusion criteria were non-peer-reviewed articles, no documented VPA use, no genetic diagnosis and lacking patient-specific information. An ADR was defined as a noxious or unintended response to VPA, where the causal relationship between VPA use and the reaction was strongly suspected.^12^ ‘Symptom-control’ referred to reported seizure or symptom reduction with VPA. The search strategy and risk of bias assessment are detailed in online supplemental information.

10.1136/bmjno-2024-000650.supp1 Supplementary data

### Survival analyses*—POLG* data set

To investigate survival between patients who had and had not been exposed to VPA, we included the MitoPhen-Expanded *POLG* data set (publications up to 01 June 2022), where patients with seizure phenotypes and without documented VPA use were coded as ‘no VPA’. The survminer R package^13^ was used to create Kaplan-Meier plots and the log-rank p value was used for testing differences in survival between groups. ‘Event’ was defined as ‘death’ for survival analysis. Time in years was calculated by age at symptom onset until age at death or age at last follow-up.

### HPO-based analyses

The OntologyX^14^ and gplots^15^ R packages were used to generate a matrix of phenotype similarity scores using the Lin similarity measure, and a heatmap displaying the hierarchically clustered scores per patient. Phenotype enrichment was explored within the *POLG* data set by grouping for VPA and no VPA exposure, and between *POLG* and non-*POLG* data sets with VPA exposure, using one-sided Fisher’s exact test adjusted with the Benjamini-Hochberg procedure. HPO terms related to liver dysfunction were excluded to avoid over-representation of VPA-related toxicity.

### Data availability

See online supplemental information. MitoPhen is accessible at www.mitophen.org.

## Results

The search strategy revealed a total of 156 patients from 90 articles with mitochondrial diseases who had reported VPA exposure (figure 1).

### Group 1: *POLG*-related mitochondrial diseases

**Figure 2:**
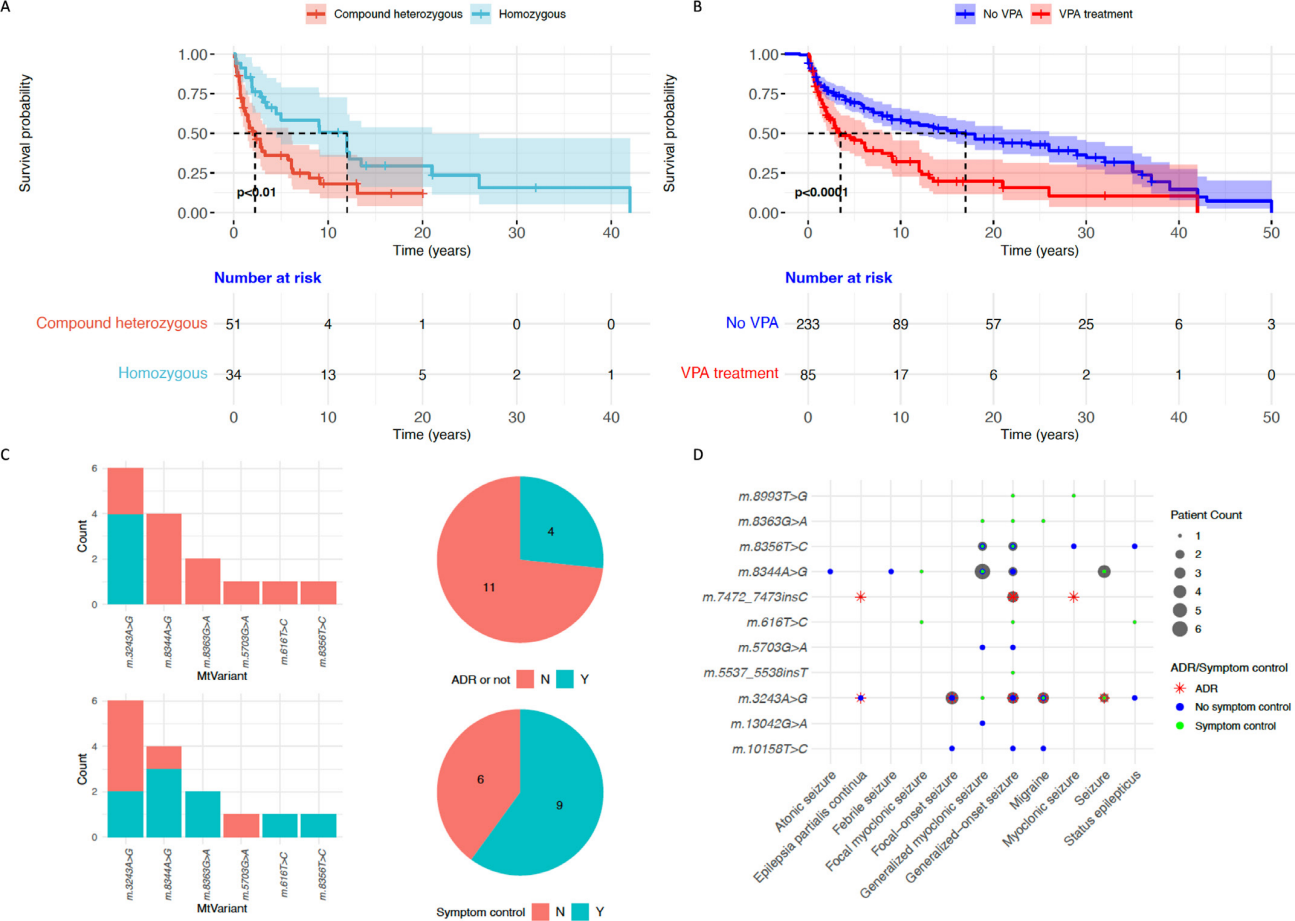
Survival in *POLG* disease and adverse reactions in mitochondrial DNA (mtDNA) disease. (A) Survival comparison in patients exposed to valproic acid (VPA) by genotype—the log-rank test revealed a significant difference between compound heterozygous and homozygous groups (p<0.01).(B) Survival comparison between documented VPA treatment and no VPA exposure groups revealed a significant difference using the log-rank test (p<0.0001). (C) VPA use by pathogenic mtDNA variant, adverse drug reaction (ADR) and reported symptom control. (D) Symptom type and mtDNA variant with counts of patients where VPA was used (displayed as grey circles). ADR and symptom control status also displayed as red asterisk or coloured dot, respectively.

Data collated from 46 articles on 102 patients with *POLG*-disease and VPA exposure (figure 1) showed ADRs in 91 patients (89%), with 87 patients (85%) reported to have hepatotoxicity. There were no reported ADRs in seven and ADR status was unknown in four patients. There was no pre-existing liver disease in 92/102 patients (90%). There were fatal outcomes in 53/102 patients (52%), with 8 being unrelated to VPA such as intractable seizures. Liver transplant was required in 15 patients, and 6 (40%) died post-transplant. Age at onset was available for 98/102 patients (96%). Survival between male and female patients was similar (p=0.7), but the lower age at onset group (0–4 years) had a lower survival compared with onset in later childhood: 5–18 years (p=0.05). All patients exposed to VPA with reported genotypes (97/102) had recessive *POLG* variants. Compound heterozygous variants were associated with worse outcomes compared with homozygous variants (p<0.01) (figure 2A).

There were 280 patients from 66 articles with recessive *POLG* variants, without documented VPA exposure included in the MitoPhen-Expanded data set. There was a lower survival in the VPA exposure group compared with no VPA use (p<0.0001, figure 2B).

### Group 2: mtDNA diseases

In the mtDNA disease group, 28 articles mentioned VPA use in 35 patients without any documented fatal ADRs. Seizure control was reported to be effective in 10/13 (77%) patients with *m.8344A>G* and presentations of myoclonic epilepsy and ragged red fibres (MERRF syndrome). Seizures were exacerbated in 3/8 (38%) *m*.*3243A>G* patients who had features suggestive of mitochondrial encephalomyopathy with lactic acidosis and stroke-like episodes (MELAS syndrome) (figure 2C, (online supplemental table S1). Patients with generalised myoclonic seizures, and/or focal myoclonic seizures, had reported good symptom control with VPA (figure 2D). There was one report of VPA being instigated for migraine in a patient successfully, although it was a dominant symptom in five published patients. There were 3/35 (9%) cases with non-fatal hepatotoxic effects including pancreatitis (online supplemental table S1).

### Group 3: other nuclear-encoded mitochondrial diseases

The search strategy for 227 nuclear genotypes of mitochondrial diseases resulted in 16 articles documenting 19 patients (figure 1). Four patients (21%) had VPA hepatotoxicity: one patient with a severe encephalopathy and epilepsy due to *WARS2* variants developed acute liver failure after commencing VPA,^16^ two patients with recessive *TWNK* variants and one with biallelic *PARS2* variants had transient elevations in hepatic transaminases. Three patients had transient symptoms where VPA was thought to be a contributing factor, however, a causal relationship was not clear. Therefore, 15/19 (79%) patients in this group had no definite VPA-induced ADRs (online supplemental table S2).

According to the Clopper-Pearson exact method,^17^ and pairwise Fisher’s exact tests with Bonferroni adjustment, VPA-induced hepatotoxicity proportions were significantly different between group 1: *POLG* disease at 0.85 (95% CI: 0.77 to 0.92), and non-*POLG* disease groups 2 and 3 at 0.13 (95% CI: 0.05 to 0.25, p<0.0001). In terms of VPA exposure, the data available for time from VPA use until ADR in 59 patients (53 diagnosed with *POLG* disease) showed that this ranged from under 1 week to 1.33 years, with a median of 1 week.

### HPO-based analyses

There were no enriched terms between the *POLG* disease data sets grouped by VPA and no VPA exposure to distinguish those who had ADRs. Hierarchical clustering of all 156 patients exposed to VPA using phenotype similarity scores showed that patients with non-*POLG* diagnoses clustered together (online supplemental figure S1A). Patients with early onset *POLG* disease had a lower survival compared with other clusters (p<0.05, (online supplemental figure S1B). The clusters resemble clinically defined phenotypic groups relating to genotypes (online supplemental figure S1C). Status epilepticus was more frequent in *POLG* disease (online supplemental figure S2), explaining why VPA was used in these patients.

## Discussion

This comprehensive systematic review used several search engines, and MitoPhen-Expanded data sets. We assessed 228 nuclear genes and 27 mtDNA genes linked to mitochondrial diseases, incorporating patient-level data for 436 patients. No published patients with dominant *POLG* variants exposed to VPA were found. Data confirm the significant risk in recessive *POLG* disease (figure 2), with fatal outcomes in 52% and ADRs in 89% of 102 patients, aligning with previous findings.^4^ In mtDNA diseases, 10/13 patients with *m.8344A>G*-induced seizures responded well to VPA with no reported ADRs. Non-*POLG* genotypes without mtDNA depletion, including *m.7472_7473insC*, *TWNK* and *PARS2* variants, showed hepatic transaminase increases in 4/54 patients, comparable to rates in non-mitochondrial diseases.^7^ The lack of natural history data on rarer genotypes meant it was difficult to attribute VPA toxicity to the fatal neurological decline of a patient with a *WARS2* diagnosis, following the resolution of their VPA-induced liver failure.^16^ The reasons for significantly different VPA-induced hepatotoxicity proportions between *POLG* (87/102) and non-*POLG* mitochondrial diseases (7/54) (p<0.0001), remain unclear.

A limitation of this review is publication bias favouring *POLG* disease data, attributed to higher status epilepticus frequency in the *POLG* group (online supplemental figure S2), necessitating additional antiseizure medications like VPA. Also, publication bias would factor in reporting of ADRs related to VPA over non-events, thereby giving a likely overestimate of ADRs. Some authors changed VPA due to perceived ADR risks in non-*POLG* disease.^18^ This contributes to uncertainty in the clinical management of mitochondrial epilepsy due to the lack of longer-term follow-up in non-*POLG* mitochondrial diseases with VPA exposure. Additionally, there were unclear causal relationships between VPA and clinical features which could be explained by the natural progression of the mitochondrial disease.^19^ Despite variability in reporting VPA dosage and time to ADR, consistent documentation of clinical features enabled survival analyses using HPO-driven clusters, revealing significantly different survival between *POLG* and non-*POLG* diseases (online supplemental figure S1). ADRs could not be predicted by phenotypic profiles in *POLG* disease.

## Conclusions

The data showed a significantly lower proportion of hepatotoxicity with VPA in non-*POLG* versus *POLG* mitochondrial diseases. Additionally, VPA resulted in reported symptom control particularly in patients with *m.8344A>G* disease, with no reported ADRs. Longer-term follow-up studies are required to define the natural history of rare nuclear genotypes of mitochondrial diseases and the role of VPA in treating patients with non-*POLG* mitochondrial diseases. We recommend VPA is contraindicated in patients carrying recessive *POLG* variants.

## Data Availability

Data are available upon reasonable request. All data relevant to the study are included in the article or uploaded as supplementary information.
