# Supplementary material for: Evidence for sodium valproate toxicity in mitochondrial diseases: a systematic analysis
Source: BMJ Neurol Open. 2024 Jun 5;6(1):e000650. doi: 10.1136/bmjno-2024-000650 (PMC11163645; doi:10.1136/bmjno-2024-000650)
Supplement: Supplementary data [file bmjno-2024-000650supp001.pdf]

Supplementary Information

Methods:

Although this review was not prospectively registered with PROSPERO [1], the search strategy is detailed below.

Search strategy

Genotype groupings: 1) *POLG*-related mitochondrial diseases, 2) mitochondrial DNA (mtDNA) diseases 3) other nuclear gene causes of mitochondrial disease as per the list of 198 'green' genes in the PanelApp mitochondrial disorders panel (downloaded 15/11/2023), including those associated with hepato-cerebral features [2]: *AARS2, ABAT, ABCB7, ACAD9, ACO2, AFG3L2, AGK, AIFM1, ANO10, APOPT1, APTX, ATAD3A, ATP5A1, ATP5D, ATP5G3, BOLA3, BTD, C12orf65, C19orf70, C1QBP, CA5A, CARS2, CHCHD10, CLPB, CLPP, COA6, COA7, COQ2, COQ4, COQ6, COQ7, COQ8A, COQ8B, COQ9, COX20, COX6A1, COX6A2, COX7B, CYC1, DARS2, DLAT, DLD, DNA2, DNAJC19, DNM1L, DNM2, EARS2, ECHS1, ELAC2, ETFDH, ETHE1, FARS2, FBXL4, FDX2, FDXR, FH, FLAD1, GARS, GDAP1, GFER, GFM1, GFM2, GLRX5, GTPBP3, HARS2, HCCS, HIBCH, HLCS, HSD17B10, HSPD1, HTRA2, IARS2, IBA57, ISCA1, ISCA2, ISCU, KARS, KIAA0391, LARS2, LIAS, LIG3, LIPT1, LIPT2, LONP1, LRPPRC, LYRM4, LYRM7, MARS2, MDH2, MECR, MFF, MFN2, MGME1, MICU1, MIPEP, MPC1, MRPL3, MRPL44, MRPS2, MRPS22, MRPS34, MSTO1, MTFMT, MTO1, MTPAP, NADK2, NARS2, NAXD, NAXE, NDUFA1, NDUFA12, NDUFA13, NDUFA4, NDUFA6, NDUFA8, NDUFA9, NDUFAF2, NDUFAF5, NDUFAF6, NDUFAF8, NDUFB10, NDUFB11, NDUFB3, NDUFB8, NDUFC2, NDUFV2, NFS1, NFU1, NSUN3, OPA1, OPA3, PARS2, PC, PDHA1, PDHB, PDHX, PDP1, PDSS1, PDSS2, PET100, PMPCA, PMPCB, PNPLA8, PNPT1, POLRMT, PPA2, PUS1, QRSL1, RARS2, RMND1, RNASEH1, RTN4IP1, SACS, SARS2, SDHA, SDHAF1, SDHB, SDHD, SERAC1, SFXN4, SLC19A2, SLC19A3, SLC25A1, SLC25A12, SLC25A19, SLC25A26, SLC25A3, SLC25A32, SLC25A38, SLC25A42, SLC25A46, SPG7, SSBP1, SURF1, TARS2, TAZ, TFAM, TIMM50, TIMM8A, TIMMDC1, TMEM126B, TOP3A, TPK1, TRIT1, TRMT10C, TRMT5, TRMU, TRNT1, TSFM, TUFM, UQCC2, UQCRB, UQCRC2, UQCRFS1, VARS2, WARS2, YARS2, DMPK*.

Groups 1 and 3 were supplemented with searches of the 'MitoPhen-Expanded' dataset which contains data (updated to June 2022) on *POLG, OPA1, TWNK,* and 72 nuclear genes causing diseases arising from deficiencies of complexes I-V of the mitochondrial electron transfer chain:

ACAD9, FOXRED1, NDUFA1, NDUFA10, NDUFA11, NDUFA12, NDUFA13, NDUFA2, NDUFA6, NDUFA8, NDUFA9, NDUFAF1, NDUFAF2, NDUFAF3, NDUFAF4, NDUFAF5, NDUFAF6, NDUFAF8, NDUFB10, NDUFB11, NDUFB3, NDUFB8, NDUFC2, NDUFS1, NDUFS2, NDUFS3, NDUFS4, NDUFS6, NDUFS7, NDUFS8, NDUFV1, NDUFV2, NUBPL, TIMMDC1, TMEM126B, SDHA, SDHAF1, SDHB, SDHD, BCS1L, CYC1, LYRM7, TTC19, UQCC2, UQCRB, UQCRC2, UQCRFS1, APOPT1, COA6, COA7, COX10, COX14, COX15, COX20, COX4I1, COX6A1, COX6A2, COX6B1, COX7B, LRPPRC, NDUFA4, PET100, SCO1, SCO2, SQOR, SURF1, TACO1, ATP5A1, ATP5D, ATP5G3, ATPAF2, TMEM70.

For group 2, we searched for 89 pathogenic mtDNA variants, as listed in MitoPhen [3], as these were manually curated to be pathogenic using the American College of Medical Genetics and Genomics guidelines [4].

Search terms used within PubMed, Ovid Embase and Ovid Medline alongside the genotypes above were: 'headache' or 'migraine' or 'seizure' or 'epilep*', and 'mitochondrial disease*' or 'mitochondrial disord*', and 'sodium valproate' or 'valproic acid'.

Ratnaike TE, *et al. BMJ Neurol Open* 2024; 6:e000650. doi: 10.1136/bmjno-2024-000650

MitoPhen-Expanded dataset: The search strategy used to compile this dataset is as per the MitoPhen database [3] – where PubMed is searched for 'gene' and 'mitochondrial disease*' or 'clinical*'. Title and abstract reviews were performed to exclude articles which did not contain patient-specific information by co-author KS.
POLG dataset: Two data collectors (co-authors NE and AL) have additionally gathered data on treatments documented. The pathogenicity of *POLG* variants was checked using American College of Medical Genetics guidelines [4], the 'Human DNA Polymerase Gamma Mutation Database' [5], and through expert consensus between co-authors KS and RH. The MitoPhen-Expanded dataset was searched for any probands with human phenotype ontology (HPO) terms relating to headache, migraine, and seizures. Additional family members diagnosed with mitochondrial disease were included if sodium valproate (VPA) treatment was mentioned.

Nuclear genotypes associated with mitochondrial complexes I-V deficiency:
PanelApp [2] was used to identify 'green' genes in these categories, and the search strategy is as above. Co-author CG collected patient-specific data including on treatments.
Note: there were no mentions of sodium valproate in the MitoPhen-Expanded *OPA1* and *TWNK* datasets, therefore we have not included these datasets in this paper.

Data extraction and review
Author TR screened the articles and collected the data. The data collected from articles documenting VPA use in patients with mitochondrial diseases included sex of patient, age at onset of symptoms, HPO terms (manually extracted), age at VPA use, age at adverse drug reaction (ADR), type of ADR, and whether the ADR was ultimately fatal. Author PFC performed a secondary review of included articles to ensure correct interpretation of data.

Risk of bias assessment summary
Author TR screened articles using the Joanna Briggs Institute case report critical appraisal tool [6]. Articles very infrequently reported dosages and frequency of administering VPA and did not report blood monitoring of VPA levels. Additional confounders such as other antiseizure medications were used in case reports, especially in the *POLG* disease datasets, which contributes to difficulty in ascertaining the true effect of VPA in ADR severity. However, the clinical presenting features and effects of suspected VPA toxicity were consistently reported across all groups. Therefore, the study was not suitable for a meta-analysis, but results could be synthesised using a phenotype-driven approach, and overall rate of ADRs could be commented on.

Literature Review Search terms:
<u>Group 1: Search strategy for *POLG*-associated mitochondrial disease</u>
Ovid Medline (1946 to September 21, 2022), Ovid Embase (1974 to September 22, 2022), PubMed (results to September 2022): 98 articles (once duplicates were removed)
MitoPhen-Expanded dataset: 12 articles
Following exclusion criteria: 46 articles included

Ovid MEDLINE(R) ALL <1946 to September 21, 2022>

**#**	**Query**	**Results from 22 Sep 2022**
1	headache*.af.	114,936
2	exp headache/	30,528
3	migraine*.af.	45,004
4	exp migraine/	30,796
5	seizure*.af.	160,206
6	exp seizure/	71,174
7	epilep*.af.	191,237
8	exp epilepsy/	122,878
9	1 or 2	114,962
10	3 or 4	45,045
11	5 or 6	163,932
12	7 or 8	193,144
13	exp mitochondrial diseases/	17,460
14	("mitochondrial DNA*" or mtDNA or "mitochondrial disease*").af.	58,981
15	13 or 14	66,818
16	("sodium valproate" or "valproic acid").af.	18,449
17	exp valproic acid/	13,639
18	polg*.af.	3,851
19	16 or 17	18,449
20	9 or 10 or 11 or 12	386,110
21	15 and 18 and 20	164
22	19 and 21	29

Embase <1974 to 2022 September 22>

#	Query	Results from 23 Sep 2022
1	headache*.af.	330,496
2	exp headache/	252,965
3	migraine*.af.	82,921
4	exp migraine/	73,997
5	seizure*.af.	274,838
6	exp seizure/	167,623
7	epilep*.af.	327,593
8	exp epilepsy/	262,676
9	1 or 2	330,496
10	3 or 4	82,924
11	5 or 6	274,838
12	7 or 8	353,670
13	exp "disorders of mitochondrial functions"/	50,704
14	("mitochondrial DNA*" or mtDNA or "mitochondrial disease*").af.	76,649
15	13 or 14	111,831
16	("sodium valproate" or "valproic acid").af.	72,136
17	exp valproic acid/	70,821
18	polg*.af.	5,166
19	16 or 17	72,136
20	9 or 10 or 11 or 12	776,563
21	15 and 18 and 20	449
22	19 and 21	105

23	"conference abstract* ".pt,st.	4,560,321
24	22 not 23	77
25	from 24 keep 1-77	77
26	from 25 keep 4,11-12,16,22,29,31,36,75	9
27	from 26 keep 1-9	9
28	from 25 keep 1-3,5-10,13-15,17-21,23-28,30,32-35,37-74,76-77	68

Search strategy with PubMed:

#	Query	Filters	Search Details	Results
**7**	(((((headache*) OR (migraine*)) OR ((seizure*) OR (epileps*))) AND (((VALPROATE*) OR (valproic acid*)) OR (VPA*))) AND ((POLG*) OR (polymerase gamma*))) AND ((mitochondrial disease*) OR (mitochondrial disord*))		("headache*"[All Fields] OR "migraine*"[All Fields] OR ("seizure*"[All Fields] OR "epileps*"[All Fields])) AND ("valproate*"[All Fields] OR ("valproic"[All Fields] AND "acid*"[All Fields]) OR "vpa"[All Fields]) AND ("polg*"[All Fields] OR (("polymerase"[All Fields] OR "polymerase s"[All Fields] OR "polymerases"[All Fields]) AND "gamma*"[All Fields])) AND ((("mitochondria"[MeSH Terms] OR "mitochondria"[All Fields] OR "mitochondrial"[All Fields] OR "mitochondrially"[All Fields] OR "mitochondrials"[All Fields]) AND "disease*"[All Fields]) OR (("mitochondria"[MeSH Terms] OR "mitochondria"[All Fields] OR "mitochondrial"[All Fields] OR "mitochondrially"[All Fields] OR "mitochondrials"[All Fields]) AND "disord*"[All Fields]))	33
**6**	((headache*) OR (migraine*)) OR ((seizure*) OR (epileps*))		"headache*"[All Fields] OR "migraine*"[All Fields] OR "seizure*"[All Fields] OR "epileps*"[All Fields]	389,191
**5**	((VALPROATE*) OR (valproic acid*)) OR (VPA*)		"valproate*"[All Fields] OR ("valproic"[All Fields] AND "acid*"[All Fields]) OR "vpa"[All Fields]	23,319
**4**	(POLG*) OR (polymerase gamma*)		"polg*"[All Fields] OR (("polymerase"[All Fields] OR "polymerase s"[All Fields] OR "polymerases"[All Fields]) AND "gamma*"[All Fields])	33,630
**3**	(seizure*) OR (epileps*)		"seizure*"[All Fields] OR "epileps*"[All Fields]	261,796
**2**	(headache*) OR (migraine*)		"headache*"[All Fields] OR "migraine*"[All Fields]	138,746
**1**	(mitochondrial disease*) OR (mitochondrial disord*)		(("mitochondria"[MeSH Terms] OR "mitochondria"[All Fields] OR "mitochondrial"[All Fields] OR "mitochondrially"[All Fields] OR "mitochondrials"[All Fields]) AND "disease*"[All Fields]) OR (("mitochondria"[MeSH Terms] OR "mitochondria"[All Fields] OR "mitochondrial"[All Fields] OR "mitochondrially"[All Fields] OR "mitochondrials"[All Fields]) AND "disord*"[All Fields])	119,014

Group 2: Search strategy for mtDNA disease

Search terms used were the same as for the *POLG* disease dataset above but each of the 89 pathogenic mtDNA variants within MitoPhen were reviewed.
Ovid Embase (1974 to June 09, 2022): 79 articles
PubMed (results to September 2022): 1 article
MitoPhen: 231 articles
Following exclusion criteria: 28 articles

Group 3: Search strategy for non-*POLG* nuclear genes

Search terms used were the same as for the *POLG* disease dataset above but each of the PanelApp genes listed above, were reviewed.

Ovid Medline (1946 to June Week 4 2022), Ovid Embase (1974 to July 01, 2022), PubMed (results updated to December 2022): 251 articles
MitoPhen-Expanded dataset: 3 articles
Following exclusion criteria: 16 articles

Results:

**Categories**	**Data**	**References**
**Demographics**	Female: 16<br>Male: 17<br>ND: 2	
**Presentations requiring VPA treatment**	Migraine: 1<br>Generalised-onset seizures: 8<br>Generalised myoclonic seizures: 18<br>Generalised-onset and generalised myoclonic seizures: 4<br>Focal-onset seizures: 4<br>Seizures (unspecified): 3	[7]<br>[7-14]<br>[15-24]<br>[23, 25-27]<br>[28-31]<br>[32, 33]
**ADRs**	Pancreatitis- *m.3243A>G*<br>Nausea and rise in liver transaminases- *m.7472_7473insC*<br>Severe Reye-like syndrome- *m. 7472_7473insC*<br>Exacerbation of seizures- *m.3243A>G* (3 patients)	[32]<br>[9]<br>[25]<br>[13, 29, 30]
**Symptom control effective**	m.616T>C – 1 patient<br>m.3243A>G – 2 patients<br>m.5537_5538insT – 1 patient<br>m.8356T>C – 1 patient<br>m.8993T>G – 1 patient<br>m.8363G>A – 3 patients<br>m.8344A>G – 10 patients	[8]<br>[21, 33]<br>[11]<br>[23]<br>[34]<br>[7, 14]<br>[10, 17-19, 24]
**Symptom control ineffective**	m.10158T>C – 1 patient<br>m.5703G>A – 1 patient<br>m.8356T>C – 1 patient<br>m.13042G>A – 1 patient<br>m.8344A>G – 3 patients<br>m.3243A>G – 5 patients	[28]<br>[26]<br>[23]<br>[22]<br>[16, 20, 27]<br>[12, 13, 29-31]

Table S1 Summary of data for published patients with mtDNA diseases and VPA exposure.

Genes	Patient details	ADR	Ref
ACAD9	1 patient: Treated at 18 months old with VPA for absence seizures, on a background of hypertrophic cardiomyopathy.	None	[35]
COQ4	1 patient: presented with a progressive neurologic disorder, with tonic-clonic seizures at age 18 years, treated successfully with VPA.	None	[36]
ECHS1	1 patient: treated with VPA for seizures in infancy, also had features resembling Leigh syndrome. VPA thought to worsen abnormal eye movements, levetiracetam also worsened features. A valine-restricted diet was noted to improve neurological features.	Possible exacerbation of seizures	[37]
ETFDH and *PHGDH*	1 patient: Childhood-onset intractable seizures, treated with VPA, in combination with lamotrigine and clonazepam, achieved full seizure control. VPA withdrawn at three years old, when developmental regression noted. Clinical status improved after the amino acid therapy commenced to treat serine deficiency.	Unclear if developmental regression associated with VPA or disease progression	[38]
NDUFV1	1 patient: Childhood-onset seizures and cerebellar ataxia, treated with VPA (at 12 months old). Death at three years old following acute metabolic acidosis and neurodegeneration.	None	[39]
OPA1	1 patient: Adult-onset occipital lobe epilepsy. VPA used in combination with several AEDs, no beneficial effects.	None	[40]
TRIT1	1 patient: Childhood-onset epilepsy triggered by febrile illness, including myoclonic epilepsy. VPA swapped to levetiracetam and clonazepam to avoid hepatotoxicity.<br>1 patient: childhood-onset generalised tonic-clonic seizures and myoclonic jerks. No improvement or worsening of seizure-control with VPA.	None	[41]<br>[42]
TWNK	2 patients: Adult-onset epilepsy including episodes of status epilepticus. VPA was initiated but discontinued after about one month, due to a rise in liver transaminases.	Raised liver transaminases	[43]
Mitochondrial aminoacyl-tRNA synthetase genes:<br>*IARS2*<br>*KARS*<br>*NARS2*<br>*PARS2*<br>*RARS2*<br>*TARS2*<br>*WARS2*	*IARS2:* 2 siblings were treated for infantile spasms with VPA, without improvement in symptoms, and a subsequent diagnosis of Leigh syndrome.<br>*KARS:* 1 patient was treated for seizures with VPA, with no further seizures noted.<br>*NARS2*: 1 patient with Leigh syndrome was treated with VPA at 1.7years of age for generalised tonic and myoclonic seizures. She developed renal Fanconi syndrome which the authors attributed to VPA as she improved biochemically when VPA was stopped. Although, renal disease is documented in the natural history.<br>*PARS2*: 1 patient with a progressive neurodegenerative disease was treated with VPA at 7.5years of age for seizures, but this was stopped within a few months due to transiently raised liver transaminases. He deteriorated with multiorgan failure and died at age 8.5years. His sibling had a similar course.<br>*RARS2*: 2 siblings were treated with VPA in combination with other antiseizure medications for myoclonus with variable response.<br>*TARS2*: 1 patient was treated with VPA for seizures characterised by 'motion arrest', no symptom control.<br>*WARS2:* 1 patient with severe developmental delay, was treated at 6years of age for a prolonged seizure with VPA. She developed acute liver failure a month later and VPA was stopped. She had severe encephalopathy despite liver enzymes normalising and died aged 6.5years.	None<br>None<br>Suspected renal Fanconi syndrome<br>Raised liver transaminases<br>None<br>None<br>Acute liver failure	[44]<br>[45]<br>[46]<br>[47]<br>[48]<br>[49]<br>[50]<br>[51]

Table S2 Articles where patients with nuclear genotypes of mitochondrial diseases have been treated with VPA.

BMJ Publishing Group Limited (BMJ) disclaims all liability and responsibility arising from any reliance placed on this supplemental material which has been supplied by the author(s)

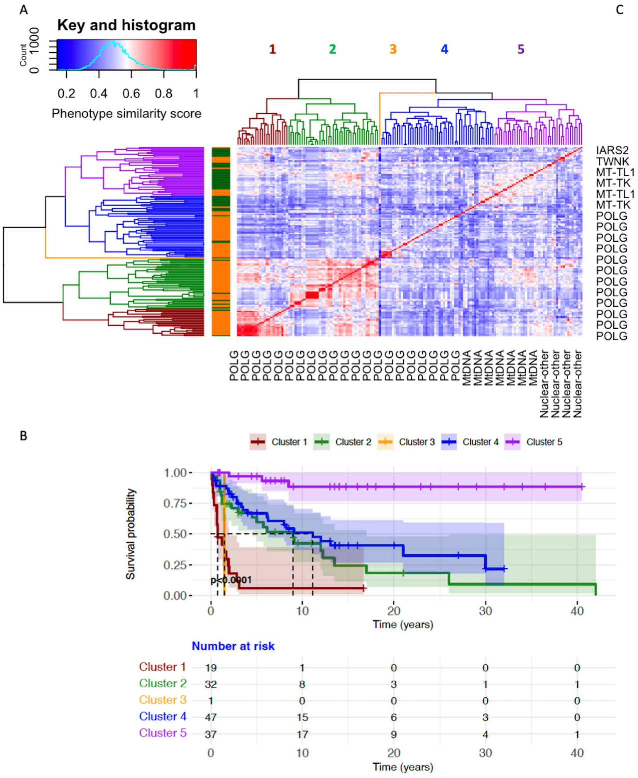

C

Cluster	HPO terms (occurring in 5 or more patients)	Frequency (%)
1	Infantile onset<br>Vomiting<br>Neurodevelopmental delay<br>Epilepsia partialis continua	16/23 (70%)<br>9/23 (39%)<br>9/23 (39%)<br>6/23 (26%)
2	Juvenile onset<br>Headache or Migraine with aura<br>Peripheral neuropathy<br>Nystagmus	12/41 (29%)<br>13/41 (32%)<br>9/41 (22%)<br>7/41 (17%)
4	Adult onset<br>Ragged-red muscle fibers<br>Generalized myoclonic seizure<br>EEG with generalized slow activity<br>EEG with spike-wave complexes<br>Dysphagia<br>Fatigue<br>Decreased activity of mitochondrial complex IV	14/51 (27%)<br>7/51 (14%)<br>7/51 (14%)<br>6/51 (12%)<br>5/51 (10%)<br>5/51 (10%)<br>5/51 (10%)<br>5/51 (10%)
5	Elevated lactate:pyruvate ratio	5/40 (13%)
All clusters	Childhood onset<br>Myoclonus or myoclonic seizure<br>Status epilepticus<br>Ataxia<br>Developmental regression<br>Focal-onset seizure<br>Generalized-onset seizure<br>Cerebral cortical atrophy	61/156 (39%)<br>64/156 (41%)<br>39/156 (25%)<br>19/156 (19%)<br>21/156 (13%)<br>19/156 (12%)<br>12/156 (8%)<br>10/156 (6%)

Figure S1 Phenotypic clustering across 156 patients with mitochondrial diseases and VPA exposure. ***A:*** *Heatmap shows hierarchical clustering of phenotype similarity scoring, causative genes are displayed on the right, and the categories of gene are shown at the bottom – indicating clustering of non-*POLG *disease in cluster 5. Cluster numbers are coloured and shown at the top of the dendrogram. The row-side colours represent documented adverse drug reactions (orange) or not (green).* ***B:*** *Survival curves for each phenotypic cluster are presented, pairwise comparisons conducted using the Log-Rank test showed a significant difference in survival between Cluster 1 and Cluster 5 against other clusters (excluding Cluster 3) (p<0.05). Confidence intervals displayed at a 95% confidence level.* ***C:*** *Table highlights the frequent HPO terms seen in each cluster (with five or more patients documented to have the term), terms found in all clusters are shown at the bottom.*

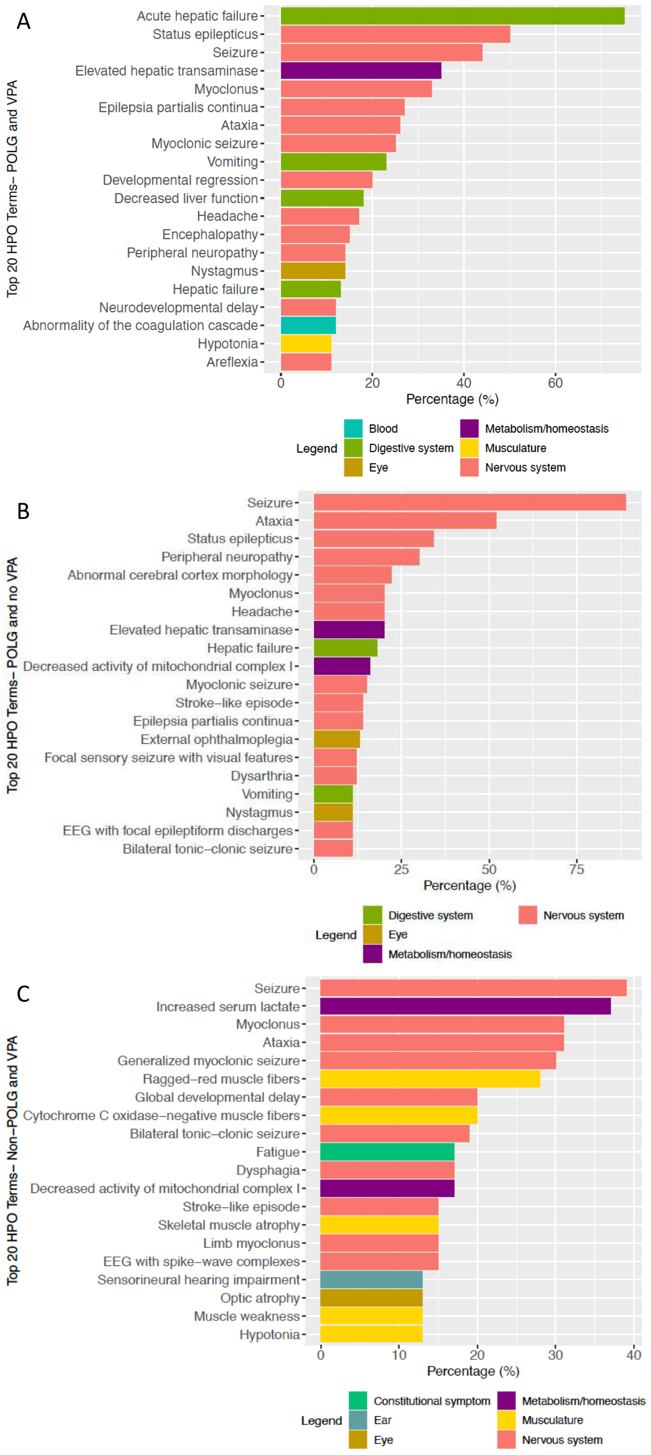

Figure S2 Frequency bar charts of top 20 human phenotype ontology (HPO) terms. ***A****: Top 20 terms in 102 patients with POLG disease and VPA exposure- status epilepticus seen in >25%.* ***B:*** *Top 20 terms in 284 patients with POLG disease and no VPA exposure- status epilepticus seen in 50%.* ***C:*** *Top 20 terms in 54 patients with non-POLG disease and VPA exposure, status epilepticus not within top 20 terms.*

References

1. Booth, A., et al., *The nuts and bolts of PROSPERO: an international prospective register of systematic reviews.* Syst Rev, 2012. **1**: p. 2.
2. Martin, A.R., et al., *PanelApp crowdsources expert knowledge to establish consensus diagnostic gene panels.* Nat Genet, 2019. **51**(11): p. 1560-1565.
3. Ratnaike, Thiloka E., et al., *MitoPhen database: a human phenotype ontology-based approach to identify mitochondrial DNA diseases.* Nucleic Acids Research, 2021. **49**(17): p. 9686-9695.
4. Richards, S., et al., *Standards and guidelines for the interpretation of sequence variants: a joint consensus recommendation of the American College of Medical Genetics and Genomics and the Association for Molecular Pathology.* Genetics In Medicine, 2015. **17**: p. 405.
5. Longley, M.J., et al., *Consequences of mutations in human DNA polymerase γ.* Gene, 2005. **354**: p. 125-131.
6. Vardell, E. and M. Malloy, *Joanna Briggs Institute: An Evidence-Based Practice Database.* Medical Reference Services Quarterly, 2013. **32**(4): p. 434-442.
7. Virgilio, R., et al., *Mitochondrial DNA G8363A mutation in the tRNA Lys gene: clinical, biochemical and pathological study.* J Neurol Sci, 2009. **281**(1-2): p. 85-92.
8. Zsurka, G., et al., *Severe epilepsy as the major symptom of new mutations in the mitochondrial tRNA(Phe) gene.* Neurology, 2010. **74**(6): p. 507-12.
9. Schuelke, M., et al., *Epilepsia partialis continua associated with a homoplasmic mitochondrial tRNA(Ser(UCN)) mutation.* Ann Neurol, 1998. **44**(4): p. 700-4.
10. Shen, C., et al., *Overlapping Leigh Syndrome/Myoclonic Epilepsy With Ragged Red Fibres in an Adolescent Patient With a Mitochondrial DNA A8344G Mutation.* Front Neurol, 2018. **9**: p. 724.
11. Tulinius, M., et al., *Leigh syndrome with cytochrome-c oxidase deficiency and a single T insertion nt 5537 in the mitochondrial tRNATrp gene.* Neuropediatrics, 2003. **34**(2): p. 87-91.
12. Chen, W.T., et al., *Adult onset MELAS Syndrome Presenting as A Mimic of Herpes Simplex Encephalitis.* Acta Neurol Taiwan, 2019. **28(2)**: p. 46-51.
13. Hsu, Y.C., et al., *Adult-onset of mitochondrial myopathy, encephalopathy, lactic acidosis, and stroke-like episodes (MELAS) syndrome presenting as acute meningoencephalitis: a case report.* J Emerg Med, 2012. **43**(3): p. e163-6.
14. Graf, W.D., et al., *Autism associated with the mitochondrial DNA G8363A transfer RNA(Lys) mutation.* J Child Neurol, 2000. **15**(6): p. 357-61.
15. Primiano, G., et al., *Drug-resistant epilepsy in MELAS: safety and potential efficacy of lacosamide.* Epilepsy Res, 2018. **139**: p. 135-136.
16. Sweeney, M.G., et al., *Mitochondrial DNA mutation underlying Leigh's syndrome: clinical, pathological, biochemical, and genetic studies of a patient presenting with progressive myoclonic epilepsy.* J Neurol Sci, 1994. **121**(1): p. 57-65.
17. Altmann, J., et al., *Expanded phenotypic spectrum of the m.8344A>G "MERRF" mutation: data from the German mitoNET registry.* J Neurol, 2016. **263**(5): p. 961-972.
18. Thompson, P.D., S.R. Hammans, and A.E. Harding, *Cortical reflex myoclonus in patients with the mitochondrial DNA transfer RNA(Lys)(8344) (MERRF) mutation.* J Neurol, 1994. **241**(5): p. 335-40.

19. Peng, Y., R. Crumley, and J.M. Ringman, *Spasmodic dysphonia in a patient with the A to G transition at nucleotide 8344 in mitochondrial DNA.* Mov Disord, 2003. **18**(6): p. 716-8.
20. Mancuso, M., et al., *MERRF syndrome without ragged-red fibers: the need for molecular diagnosis.* Biochem Biophys Res Commun, 2007. **354**(4): p. 1058-60.
21. Fabrizi, G.M., et al., *The A to G transition at nt 3243 of the mitochondrial tRNALeu(UUR) may cause an MERRF syndrome.* J Neurol Neurosurg Psychiatry, 1996. **61**(1): p. 47-51.
22. Slawek, J., et al., *Mitochondrial encephalopathy in a patient with a 13042G>A de novo mutation.* J Clin Pathol, 2012. **65**(12): p. 1147-9.
23. Nakamura, M., et al., *MERRF/MELAS overlap syndrome: a double pathogenic mutation in mitochondrial tRNA genes.* J Med Genet, 2010. **47**(10): p. 659-64.
24. Ohtsuka, Y., et al., *Myoclonus epilepsy with ragged-red fibers: a clinical and electrophysiologic follow-up study on two sibling cases.* J Child Neurol, 1993. **8**(4): p. 366-72.
25. Jaksch, M., et al., *Progressive myoclonus epilepsy and mitochondrial myopathy associated with mutations in the tRNA(Ser(UCN)) gene.* Ann Neurol, 1998. **44**(4): p. 635-40.
26. Fu, J., et al., *Broadening the phenotype of m.5703G>A mutation in mitochondrial tRNAAsn gene from mitochondrial myopathy to myoclonic epilepsy with ragged red fibers syndrome.* Chin Med J (Engl), 2019. **132**(7): p. 865-867.
27. Kimura, S., et al., *Myoclonic epilepsy with ragged-red fibers without increased lactate levels.* Pediatr Neurol, 2009. **41**(1): p. 46-8.
28. Vodopivec, I., et al., *Mitochondrial Encephalopathy and Optic Neuropathy Due to m.10158 MT-ND3 Complex I Mutation Presenting in an Adult Patient: Case Report and Review of the Literature.* Neurologist, 2016. **21**(4): p. 61-5.
29. Lam, C.W., et al., *Mitochondrial myopathy, encephalopathy, lactic acidosis and stroke-like episodes (MELAS) triggered by valproate therapy.* Eur J Pediatr, 1997. **156**(7): p. 562-4.
30. Lin, C.M. and P. Thajeb, *Valproic acid aggravates epilepsy due to MELAS in a patient with an A3243G mutation of mitochondrial DNA.* Metab Brain Dis, 2007. **22**(1): p. 105-9.
31. Cesaroni, E., et al., *Mitochondrial encephalomyopathy lactic acidosis and strokelike episodes mimicking occipital idiopathic epilepsy.* Pediatr Neurol, 2009. **41**(2): p. 131-4.
32. Dougherty, F.E., S.G. Ernst, and J.R. Aprille, *Familial recurrence of atypical symptoms in an extended pedigree with the syndrome of mitochondrial encephalomyopathy, lactic acidosis, and stroke-like episodes (MELAS).* J Pediatr, 1994. **125**(5 Pt 1): p. 758-61.
33. Sun, X., et al., *MELAS and macroangiopathy: A case report and literature review.* Medicine (Baltimore), 2018. **97**(52): p. e13866.
34. Jung, J., et al., *NARP mitochondriopathy: an unusual cause of progressive myoclonic epilepsy.* Neurology, 2007. **68**(17): p. 1429-30.
35. Collet, M., et al., *High incidence and variable clinical outcome of cardiac hypertrophy due to ACAD9 mutations in childhood.* Eur J Hum Genet, 2016. **24**(8): p. 1112-6.
36. Mero, S., et al., *New pathogenic variants in COQ4 cause ataxia and neurodevelopmental disorder without detectable CoQ(10) deficiency in muscle or skin fibroblasts.* J Neurol, 2021. **268**(9): p. 3381-3389.

37. Muntean, C., et al., *Pathogenic Biallelic Mutations in ECHS1 in a Case with Short-Chain Enoyl-CoA Hydratase (SCEH) Deficiency-Case Report and Literature Review.* Int J Environ Res Public Health, 2022. **19**(4).
38. Ali, A., et al., *Characterization of ETFDH and PHGDH Mutations in a Patient with Mild Glutaric Aciduria Type II and Serine Deficiency.* Genes (Basel), 2021. **12**(5).
39. Bénit, P., et al., *Large-scale deletion and point mutations of the nuclear NDUFV1 and NDUFS1 genes in mitochondrial complex I deficiency.* Am J Hum Genet, 2001. **68**(6): p. 1344-52.
40. Nass, R.D., et al., *Retinoencephalopathy with occipital lobe epilepsy in an OPA-1 mutation carrier.* Seizure, 2019. **66**: p. 1-3.
41. Takenouchi, T., et al., *Noninvasive diagnosis of TRIT1-related mitochondrial disorder by measuring i(6) A37 and ms(2) i(6) A37 modifications in tRNAs from blood and urine samples.* Am J Med Genet A, 2019. **179**(8): p. 1609-1614.
42. Yıldırım, M., et al., *A Case of Combined Oxidative Phosphorylation Deficiency 35 Associated with a Novel Missense Variant of the TRIT1 Gene.* Mol Syndromol, 2022. **13**(2): p. 139-145.
43. Lönnqvist, T., et al., *Recessive twinkle mutations cause severe epileptic encephalopathy.* Brain, 2009. **132**(6): p. 1553-1562.
44. Takezawa, Y., et al., *Novel IARS2 mutations in Japanese siblings with CAGSSS, Leigh, and West syndrome.* Brain Dev, 2018. **40**(10): p. 934-938.
45. Ardissone, A., et al., *KARS-related diseases: progressive leukoencephalopathy with brainstem and spinal cord calcifications as new phenotype and a review of literature.* Orphanet J Rare Dis, 2018. **13**(1): p. 45.
46. Tanaka, R., et al., *Novel NARS2 variant causing leigh syndrome with normal lactate levels.* Hum Genome Var, 2022. **9**(1): p. 12.
47. Sofou, K., et al., *The phenotypic variability and natural history of NARS2 associated disease.* Eur J Paediatr Neurol, 2021. **31**: p. 31-37.
48. Ciara, E., et al., *Clinical and molecular characteristics of newly reported mitochondrial disease entity caused by biallelic PARS2 mutations.* Journal of Human Genetics, 2018. **63**(4): p. 473-485.
49. Mathew, T., et al., *Expanding spectrum of RARS2 gene disorders: Myoclonic epilepsy, mental retardation, spasticity, and extrapyramidal features.* Epilepsia Open, 2018. **3**(2): p. 270-275.
50. Li, X., et al., *Novel compound heterozygous TARS2 variants in a Chinese family with mitochondrial encephalomyopathy: a case report.* BMC Med Genet, 2020. **21**(1): p. 217.
51. Vantroys, E., et al., *Severe hepatopathy and neurological deterioration after start of valproate treatment in a 6-year-old child with mitochondrial tryptophanyl-tRNA synthetase deficiency.* Orphanet J Rare Dis, 2018. **13**(1): p. 80.
